# MicroRNA-381 Negatively Regulates TLR4 Signaling in A549 Cells in Response to LPS Stimulation

**DOI:** 10.1155/2015/849475

**Published:** 2015-11-24

**Authors:** Zhihao Xu, Dapeng Dong, Xiaofei Chen, Huaqiong Huang, Shenglan Wen

**Affiliations:** ^1^The Fourth Affiliated Hospital of Zhejiang University School of Medicine, No. 1 City Mall Street, Yiwu, Zhejiang 322000, China; ^2^The Second Affiliated Hospital of Zhejiang University School of Medicine, Jiefang Road 88, Hangzhou, Zhejiang 310009, China

## Abstract

It is widely reported that miR-381 is dysregulated in various tumors. However, the specific role of miR-381 in respiratory infections has not been reported. To probe this role, A549 cells were pretreated with 1 *μ*g/mL LPS for 24 h. The level of miR-381 was detected using RT-qPCR. The expression of proinflammatory cytokines was determined using an ELISA kit and western blotting. Bioinformatics analysis was used to predict the target genes of miR-381, and a luciferase reporter assay was used to validate the expression of the target genes. miR-381 expression was increased in A549 cells treated with LPS, which is a ligand of TLRs. Further study revealed that the overexpression of miR-381 increased the activity of NF-*κ*B signaling, thereby increasing the expression of IL-6, TNF*α*, and COX-2. Further study revealed that I*κ*B*α* was a target gene of miR-381. The upregulation of miR-381 under LPS stimulation contributes to respiratory infections mainly by targeting I*κ*B*α*.

## 1. Introduction

As important pattern recognition receptors, Toll-like receptors (TLRs) are generally expressed in antigen-presenting cells, including macrophages and dendritic cells [1]. TLRs can recognize pathogen-associated molecular patterns (PAMPs) such as lipopolysaccharides (LPS) [2]. Through the TLR-mediated recognition of PAMPs, the innate immune response can be rapidly activated, which plays a key role in the host defense against microorganisms [3]. It has been suggested that the early immune inflammatory response is initiated through the activation of NF-*κ*B signaling [4]. Then, the p65/p50 heterodimer translocates to the nucleus and triggers the transcription of many proinflammatory cytokines [4]. In normal cells, transcription and NF-*κ*B signaling activity are under strict regulation.

MicroRNAs (miRNAs, miRs) are small noncoding RNAs that are widely expressed in different tissues [5]. Through incomplete binding of the 3′ untranslated region (3′UTR), miRNAs repress the expression of target genes. It has been suggested that miRNAs are widely involved in a series of pathological processes such as cell differentiation, immunity, and inflammation [6]. Recent studies have demonstrated a direct correlation between miRNAs and innate immune response [7–9]. For instance, miR-200b and miR-200c are reported to repress target genes that are involved in immune response [9].

In this study, we focused on miR-381, which is found to be dysregulated in various tumors [10, 11]. For instance, miR-381 is highly upregulated in glioblastoma and has been indicated as a possible target for glioblastoma multiforme therapy [10]. Additionally, in 786-O cells, miR-381 enhances the sensitivity to 5-fluorouracil by targeting WEE1 [11]. Furthermore, in lung adenocarcinomas, it was found that the decreased level of miR-381 may contribute to metastatic potential by targeting ID1 [12]. However, little research has been conducted on the potential role of miR-381 in the innate immune response. Here, we first found that miR-381 expression was obviously increased in A549 cells treated with LPS, a ligand that stimulates TLR4. Further study revealed that miR-381 overexpression increased the activity of NF-*κ*B signaling, thereby increasing the expression of IL-6, TNF*α*, and COX-2. A luciferase reporter assay revealed that I*κ*B*α* was a target gene of miR-381.

## 2. Materials and Methods

### 2.1. Cell Culture, Treatment, and Transfection

Immortalized human bronchial epithelial cells (BEAS-2B) and A549 cells were purchased from the American Type Culture Collection (Manassas, VA, USA) and were cultured in Dulbecco's modified Eagle's medium (DMEM) supplemented with 10% fetal bovine serum. Primary human alveolar type 2 cells (AT-II) that have been immortalized with SV40 were purchased from Applied Biological Materials, Inc. (Richmond, BC, Canada) and cultured in PriGrow III medium (ABM, Inc.) supplemented with 10% fetal bovine serum in collagen-coated flasks. All cells were cultured at 37°C in 5% CO_2_, as directed by the supplier.

miR-381/NC mimics and miR-381/NC inhibitors were obtained from GenePharma (Shanghai, China). Cells were transfected with RNAs using the HiPerFect transfection reagent according to the manufacturer's instructions (Qiagen, Germany).

### 2.2. MTT Assay

To determine cell viability, an MTT assay was conducted. Briefly, A549 cells were plated in 96-well culture plates. The cells were then cultured in serum-free DMEM overnight, after the cells reached 70–80% confluence. After LPS treatment, the cells were cultured in DMEM (with 10% FBS) containing 0.5 mg/mL MTT for 4 h. Then, DMSO was added to dissolve the blue formazan product, and cell viability was determined by measuring the absorbance at a wavelength of 550 nm.

### 2.3. Treatment with LPS in A549 Cells

The miR-381 mimics or NC were transfected into A549 cells for 24 h with or without 1 *μ*g/mL LPS for 6 h before harvesting. The level of miR-381 was detected using RT-qPCR. The protein levels of I*κ*B*α* were detected using western blotting.

### 2.4. Quantification of miRNAs and mRNAs

Total RNA was extracted from the cells using the TRIzol reagent (Invitrogen, Life Technologies, Carlsbad, CA, USA). RNA quality was determined using the OD_260_/OD_280_ method. The stem-loop method was applied to study the expression of mature miRNA. The total mRNA was transcribed using M-MLV (M1701, Promega Corporation, Madison, WI, USA). RT-PCR was performed as previously described [13]. The relative miR-381 content was normalized to the level of U6, and the mRNA level was normalized to GAPDH.

### 2.5. Luciferase Reporter Assay

The 3′ untranslated region (3′UTR) of I*κ*B*α* was cloned into the pmirGLO plasmid (pmirGLO-I*κ*B*α*), and the empty vector was used as a control. Another luciferase reporter system, which contained the cDNA of NF-*κ*B, was constructed using the pGL3 plasmid. The plasmids were cotransfected with miR-381 mimics or the NC into HEK293T cells for 48 h at a final concentration of 100 nM. Transfection was conducted using the VigoFect transfection reagent according to the manufacturer's instructions (Beijing, China). A Dual-Luciferase Reporter System was used to determine the relative luciferase activity (Promega, Madison, WI, USA).

### 2.6. Western Blot Analysis

Total cell lysates were extracted from the cells using RIPA buffer (Solarbio, Beijing, China) and were then subjected to 12% SDS-PAGE. Then, the proteins were transferred onto a PVDF membrane at 200 mA for 3 h. The membranes were soaked with 8% milk and washed with PBST three times (10 min/time). The membranes were then incubated with the following primary antibodies: I*κ*B*α* (44D4) Rabbit mAb#4812 (Cell Signaling Technology, Beverly, MA, USA), Phospho-NF-*κ*B p65 (Ser536) (93H1) Rabbit mAb3033 (Cell Signaling Technology, Beverly, MA, USA), NF-*κ*B p65 (D14E12) XP Rabbit mAb8242 (Cell Signaling Technology, Beverly, MA, USA), and GAPDH (D16H11) XP Rabbit mAb (Cell Signaling Technology, Beverly, MA, USA). After incubation for 2 h at room temperature (RT), the membranes were washed with PBST three times again. Then, they were incubated with their respective secondary antibodies for another 2 h. The relative band density was determined using the Tanon 5200 Multifunctional Imaging System (Beijing, China) with the ECL Western Blotting Substrate Kit (Millipore, Billerica, MA, USA). GAPDH was used as an internal control.

### 2.7. Determination of Cytokines

To determine the secretion levels in the cell medium of cytokines, including IL-6, TNF*α*, and COX-2, ELISA kits (R&D Systems, Minneapolis, MN, USA) were used.

### 2.8. Statistical Analysis

All data were obtained from three independent experiments. The data are expressed as the mean ± SD and were compared between two groups using Student's *t*-test. Statistical significance was defined as a *P* value of <0.05.

## 3. Results

### 3.1. LPS Treatment Enhanced miR-381 Levels in a Dose- and Time-Dependent Manner

**Figure 1:**
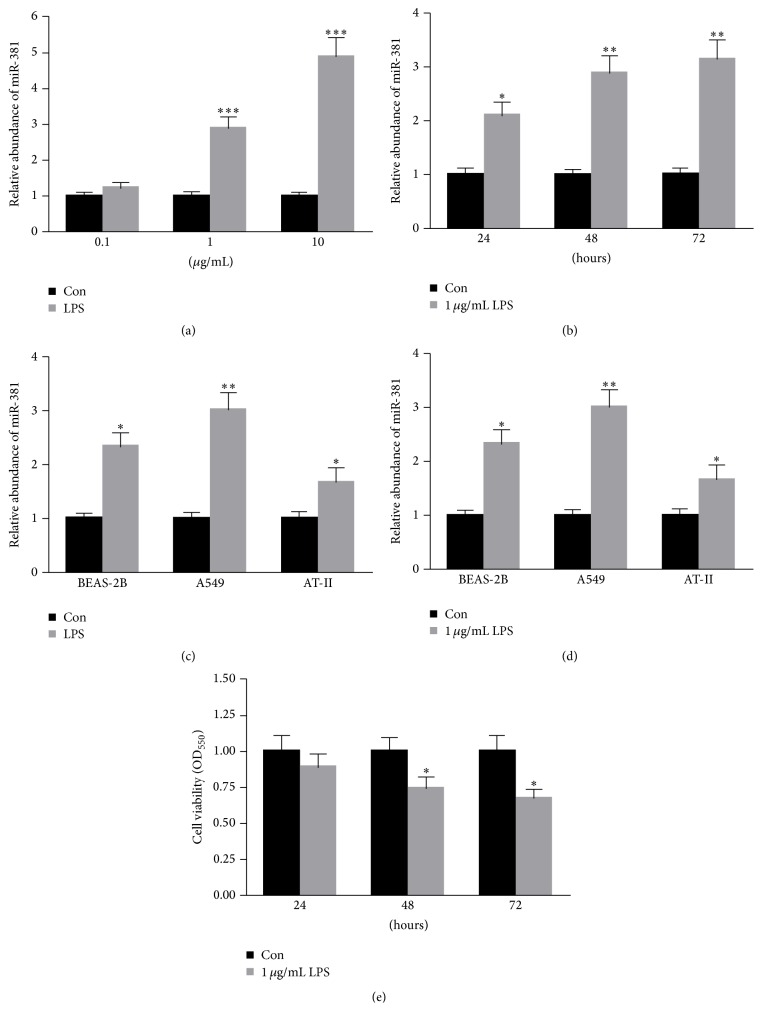
LPS treatment enhanced the level of miR-381 in a dose- and time-dependent manner. An MTT assay was conducted for A549 cells exposed to (a) 0.1, 1, and 10 *μ*g/mL of LPS for 48 h or (b) treated with 1 *μ*g/mL of LPS for 24 h, 48 h, and 72 h. (c) Pretreatment with 1 *μ*g/mL of LPS for 48 h significantly enhanced the expression of miR-381 in BEAS-2B, A549, and AT-II cells. Cell viability was determined for A549 cells exposed to (d) 0.1, 1, and 10 *μ*g/mL of LPS for 48 h or (e) treated with 1 *μ*g/mL of LPS for 24 h, 48 h, and 72 h. *n* = 3 independent experiments. ^*∗*^ *P* < 0.05, ^*∗∗*^ *P* < 0.01.

To determine whether miR-381 participated in TLR signaling, A549 cells were treated with LPS at different concentrations at different time points. As shown in Figure 1(a), when A549 cells were exposed to 0.1, 1, and 10 *μ*g/mL of LPS for 48 h, the level of miR-381 significantly increased. Moreover, treatment with 1 *μ*g/mL LPS for 24 h, 48 h, and 72 h also obviously enhanced the expression of miR-381 in A549 cells. We also explored the cell viability with these same treatment conditions. We determined the expression of miR-381 in response to 1 *μ*g/mL of LPS for 48 h in BEAS-2B, A549, and AT-II cells. It was found that pretreatment with 1 *μ*g/mL of LPS for 48 h significantly enhanced the expression of miR-381 in BEAS-2B, A549, and AT-II cells (Figure 1(c)). More importantly, an obvious upregulation of miR-381 was detected in A549 cells treated with LPS (Figure 1(c)). Thus, A549 cells were further used in the remaining experiments. As shown in Figure 1(d), treatment with 10 *μ*g/mL of LPS decreased cell viability, while only a slight decrease in viability can be detected in treatments with 0.1 and 1 *μ*g/mL of LPS. Furthermore, 1 *μ*g/mL of LPS did not significantly decrease cell viability at 24 h (Figure 1(e)). Thus, in this study, 1 *μ*g/mL of LPS was used to treat A549 cells for 24 h.

### 3.2. I*κ*B*α* Is a Target Gene of miR-381

**Figure 2:**
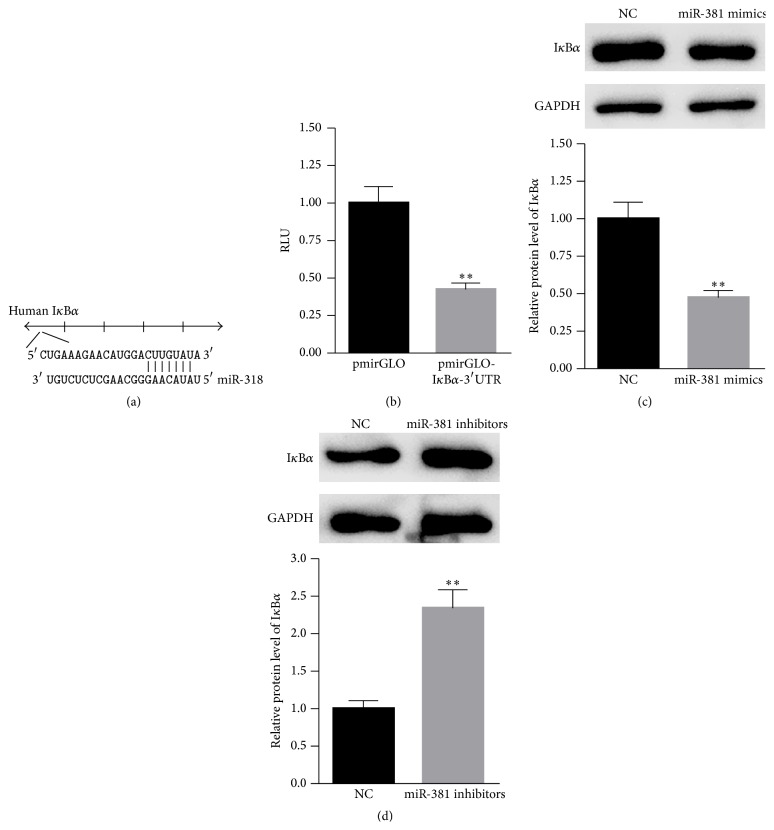
I*κ*B*α* is a target gene of miR-381. (a) Bioinformatics predictions indicate the potential binding sites of miR-381 in the 3′UTR of I*κ*B*α*. (b) The luciferase activity significantly decreased in cells cotransfected with pmirGLO-I*κ*B*α*-3′UTR and miR-381 mimics. (c) The overexpression of miR-381 decreased the protein level of I*κ*B*α*. (d) The inhibition of miR-381 increased the expression of I*κ*B*α*. *n* = 3 independent experiments. ^*∗*^ *P* < 0.05, ^*∗∗*^ *P* < 0.01.

Bioinformatics predictions indicate that I*κ*B*α* may be a possible target gene of miR-381 (http://www.TargetScan.com) (Figure 2(a)). Thus, the 3′UTR was cloned into the pmirGLO plasmid. As shown in Figure 2(b), the overexpression of miR-381 significantly decreased the luciferase activity of pmirGLO-I*κ*B*α*-3′UTR. Furthermore, western blot analysis revealed that the overexpression of miR-381 obviously decreased the protein level of I*κ*B*α* (Figure 2(c)). In comparison, the inhibition of miR-381 enhanced the expression of I*κ*B*α* (Figure 2(d)). These data suggest that I*κ*B*α* is a target gene of miR-381.

### 3.3. I*κ*B*α* Suppresses NF-*κ*B Activation and IL-6 Production

**Figure 3:**
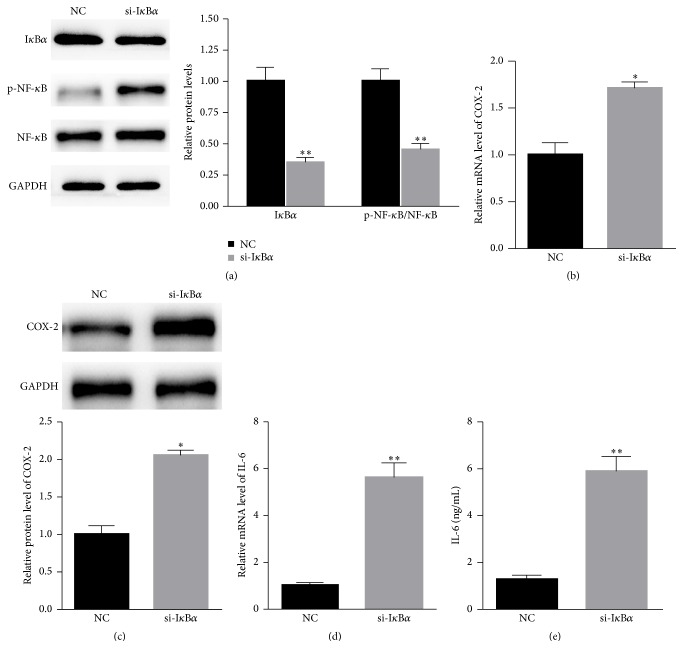
I*κ*B*α* suppresses NF-*κ*B activation and IL-6 production. (a) NF-*κ*B activation was enhanced in A549 cells transfected with si-I*κ*B*α*. The inhibition of I*κ*B*α* significantly enhanced the (b) mRNA and (c) protein level of COX-2 as detected by RT-PCR or western blot. (d) The mRNA level and (e) protein level of IL-6 also increased in A549 cells treated with si-I*κ*B*α* as analyzed by RT-PCR and an ELISA kit, respectively. *n* = 3 independent experiments. ^*∗*^ *P* < 0.05, ^*∗∗*^ *P* < 0.01.

To explore the role of I*κ*B*α* on NF-*κ*B activation, specific siRNA targeting I*κ*B*α* was transfected into A549 cells. As shown in Figure 3(a), the knockdown of I*κ*B*α* obviously suppressed the expression of I*κ*B*α*. More importantly, p-NF-*κ*B and NF-*κ*B expression increased. We further studied the expression of NF-*κ*B regulated cytokines. As shown in Figures 3(b) and 3(c), the inhibition of I*κ*B*α* significantly enhanced the mRNA and the protein level of COX-2. Moreover, the mRNA level and the protein level of IL-6 also increased in A549 cells treated with si-I*κ*B*α* (Figures 3(d) and 3(e)).

### 3.4. miR-381 Prompts NF-*κ*B Activation and Increases Inflammatory Gene Expression

**Figure 4:**
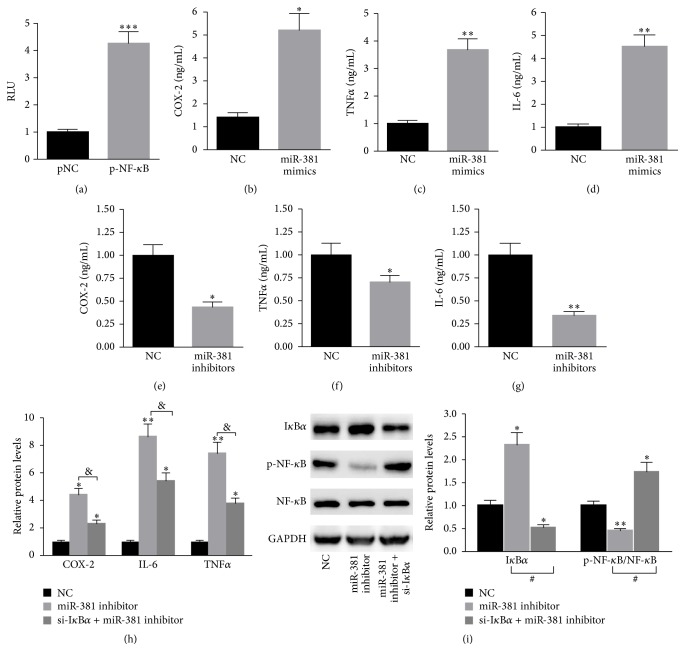
miR-381 prompts NF-*κ*B activation and increases inflammatory gene expression, partially through targeting I*κ*B*α*. (a) The overexpression of miR-381 significantly increased the luciferase activity of NF-*κ*B. Transfection of A549 cells with miR-381 mimics markedly enhanced the level of (b) COX-2, (c) TNF*α*, and (d) IL-6. The inhibition of miR-381 obviously reduced the level of (e) COX-2, (f) TNF*α*, and (g) IL-6. (h) The upregulation of COX-2, IL-6, and TNF*α* induced by LPS treatment could be partially reversed through the inhibition of miR-381 expression. (i) The miR-381 inhibitor failed to suppress NF-*κ*B activation in A549 cells transfected with si-I*κ*B*α*. *n* = 3 independent experiments. ^*∗*^ *P* < 0.05, ^*∗∗*^ *P* < 0.01.

Further studies were designed to explore the role of miR-381 in the TLR-mediated inflammatory response. We constructed a luciferase reporter system which contained the cDNA of NF-*κ*B. The overexpression of miR-381 significantly increased the luciferase activity of NF-*κ*B (Figure 4(a)). In addition, transfection of miR-381 mimics into A549 cells markedly enhanced the levels of COX-2, TNF*α*, and IL-6 (Figures 4(b), 4(c), and 4(d)). In comparison, the inhibition of miR-381 obviously reduced the levels of COX-2, TNF*α*, and IL-6 (Figures 4(e), 4(f), and 4(g)). Furthermore, we checked the kinetics of NF-*κ*B activation and the cytokine gene expression in response to LPS treatment followed by the inhibition of miR-381 in A549 cells. As shown in Figure 4(h), the upregulation of COX-2, IL-6, and TNF*α* induced by LPS treatment could be partially reversed through the inhibition of miR-381 expression. More importantly, the miR-381 inhibitor failed to suppress NF-*κ*B activation in A549 cells transfected with si-I*κ*B*α* (Figure 4(i)). These data indicated that miR-381 enhances NF-*κ*B activation through targeting I*κ*B*α*.

## 4. Discussion

In the present study, we first demonstrated that the expression of miR-381 was increased in A549 cells treated with LPS, a TLR activator. Further study identified that I*κ*B*α* is a target gene of miR-381, thereby regulating the activation of NF-*κ*B signaling and the production of proinflammatory factors.

It has been widely reported that miR-381 is involved in cancer progression and drug resistance [10, 11, 14, 15]. For instance, miR-381 was found to target WEE1, thereby regulating cell proliferation in renal cancer cells [11]. In addition, miR-381 was reported to suppress the expression of ID1 in lung adenocarcinoma [12]. Also, through targeting LRRC4, miR-381 regulates glioma growth [15]. However, little research has been conducted on the correlation between miR-381 and NF-*κ*B activation. In this study, we explored the role of miR-381 in respiratory infections. Firstly, we found that treatment with LPS significantly enhanced miR-381 expression in a time- and dose-dependent manner. Then, we predicted the possible target gene of miR-381 and found that I*κ*B*α* was a possible target gene. Western blot analysis and a luciferase assay validated that miR-381 could bind to the 3′UTR of miR-381.

As the first line of defense, the innate immunity plays a key role in the prevention of the invasion of pathogens [16]. Toll-like receptors can directly sense these outside invaders from microbial components and then trigger the innate immune response [17]. All TLR signaling activates NF-*κ*B signaling, which then initiates the expression of inflammatory cytokines [18, 19]. The activation of NF-*κ*B signaling requires the phosphorylation and degradation of inhibitor *κ*B (I*κ*B) proteins, including two kinases, I*κ*B*α* and I*κ*B*β*. NF-*κ*B is composed of two subunits, p65 and p50 [20, 21]. Generally, I*κ*B*α* can bind p65 in the cytoplasm, thereby suppressing its translocation into the nucleus. The NF-*κ*B responsive cytokine genes include TNF*α*, IL-6, and COX-2 [22]. In the present study, we found that I*κ*B*α* is a target gene of miR-381 in A549 cells. We propose that miR-381 may be involved in respiratory infections. As expected, we found that enhanced miR-381 expression significantly upregulated the expression of TNF*α*, IL-6, and COX-2.

Taken together, our findings identify a proinflammatory role of miR-381 in respiratory infections through TLR signaling. We also found that I*κ*B*α* is a direct target gene for miR-381, which suppresses the activation of NF-*κ*B signaling. Given the important role of miR-381 in LPS-induced respiratory infections, our study may shed light on new treatment methods for respiratory infections through the inhibition of miR-381.
